# Measuring the Response Performance of U.S. States against COVID-19 Using an Integrated DEA, CART, and Logistic Regression Approach

**DOI:** 10.3390/healthcare9030268

**Published:** 2021-03-03

**Authors:** Yuan Xu, Yong Shin Park, Ju Dong Park

**Affiliations:** 1 School of Maritime Economics and Management, Dalian Maritime University, 1 Linghai Road, Dalian 116026, China; yuan.xu0109@gmail.com; 2 Department of Marketing, Operations, and Analytics, Bill Munday School of Business, St. Edward’s University, 3001 South Congress, Austin, TX 78704, USA; 3 Department of Maritime Police and Production System, College of Marine Science, Gyeongsang National University, 2-6 Tongyeonghaean-ro, Tongyeong-si 53064, Gyeongsangnam-do, Korea

**Keywords:** COVID-19, DEA, classification and regression tree, logistic regression, machine learning

## Abstract

Measuring the U.S.’s COVID-19 response performance is an extremely important challenge for health care policymakers. This study integrates Data Envelopment Analysis (DEA) with four different machine learning (ML) techniques to assess the efficiency and evaluate the U.S.’s COVID-19 response performance. First, DEA is applied to measure the efficiency of fifty U.S. states considering four inputs: number of tested, public funding, number of health care employees, number of hospital beds. Then, number of recovered from COVID-19 as a desirable output and number of confirmed COVID-19 cases as a undesirable output are considered. In the second stage, Classification and Regression Tree (CART), Boosted Tree (BT), Random Forest (RF), and Logistic Regression (LR) were applied to predict the COVID-19 response performance based on fifteen environmental factors, which were classified into social distancing, health policy, and socioeconomic measures. The results showed that 23 states were efficient with an average efficiency score of 0.97. Furthermore, BT and RF models produced the best prediction results and CART performed better than LR. Lastly, urban, physical inactivity, number of tested per population, population density, and total hospital beds per population were the most influential factors on efficiency.

## 1. Introduction

On 11 February 2020, the United States was the next in a series of countries to acknowledge community spread of a deadly virus which would eventually be named COVID-19. The first COVID-19 patient was reported in the USA was in Washington State, and then the coronavirus quickly spread throughout the entire state. COVID-19 has since resulted in a high number of deaths and the confirmed cases in the United States. People and governments in the USA have been challenged by COVID-19 and its consequences [1]. Social distancing and personal protective measures, including handwashing, wearing masks and gloves became the primary means of controlling the spread of COVID-19. There are many questions, such as “What will be the short-term and long-term consequences of COVID-19?” “How efficiently have U.S. governments responded to the COVID-19 pandemic?” And more importantly, “what factors might impact efficiency?” Efficiency measures require thoughtful consideration of the factors that affect the fast spread of COVID-19. Singh and Adhikari [2] and Liu, Chen, Lin, and Han [3] found that population density and percentage of elderly significantly affect the spread of COVID-19. It is imperative to control the spread of the COVID-19 with aggressive action in the U.S. Every state must plan for faster access to existing or newer testing centers and allocate enough health care employees for people who need emergent treatment.

The degree of infection varies from country to country, and the way to control infections varies according to national conditions. How the global pandemic s contained is the most concerning problem worldwide. Therefore, it is of striking significance to predict the pandemic trends of infection worldwide. The U.S. government has taken several actions to respond to the COVID-19 pandemic, such as social distancing measures, including stay-at-home duration, non-essential business closures, and bans on large gatherings. The second directive is health policy actions, including mask mandates, public funding, expanded access to telehealth services, vaccine given status, etc. The third mitigation measure includes health care provider capacity, such as health care employees and hospital beds [4]. There are significant challenges due to unprecedented disease outbreaks, which negatively affect society as a whole and the efficiency of the COVID-19 response management in each state. Therefore, it is critical to predict the efficiency of pandemic response in the USA.

According to Correia et al. [5], the 1918 Flu had substantial variation among U.S. states in terms of the speed and aggressiveness of its spread. The study found a direct linkage between the speed and aggressiveness of interventions and containment of the virus and death rates. However, different results were found by Eichenbaum et al. [6]. They argued that there was a linkage between the virus spread and interactions with various economic decisions. The more people refrained from social contact, the higher impact it had on the pandemic.

It is important that every state health system put an upper boundary on the number of patients who can receive treatment. According to Gourinchas, [7] “with a two percent case fatality rate baseline for overwhelmed health systems and 50 percent of the world population infected, 76 million people or one percent of the world population would die.”

Measuring the efficiency of decision-making units (DMUs) such as hospitals, departments, and states using multiple inputs and producing multiple outputs is a complex part of the performance measure study. Banker et al. [8] introduced a non-parametric method to measure the technical efficiency of a set of multiple comparable DMUs, called data envelopment analysis (DEA). DEA has been used widely to analyze efficiency in the health care sector. We found several papers that used DEA to measure the health system’s efficiency in the midst of the COVID-19 pandemic, although many papers evaluate the health care system within countries and between counties [9,10,11,12].

Jouzdani et al. [10] are the first authors to perform an exploratory analysis of the global fight against COVID-19. This study used statistical analysis and visualization techniques to distinguish the temporal confirmed case, death, and recovered cases. Multiple countries are compared and the United States had significantly higher spread of COVID-19 than other countries. Another study by Shirouyehzad et al. [11] performed DEA analysis to measure countries’ efficiency affected by COVID-19. They used population density and health care infrastructure datasets to perform DEA analysis. Two-step analyses are performed to estimate efficiency. In the first step, technical efficiency scores are estimated based on the number of confirmed cases. In the second step, the total number of confirmed cases, the death rates, and recovered cases are considered to measure the countries’ efficiency of medical treatment.

Literature reviews analyzed the efficiency based on the ratio of a measure of some quality of life for output variable and health care resources or health care expenditure for input variables. This research study is similar to those, but considers different aspects using the undesirable output variable. Previous studies have primarily focused on identifying efficiency and lacked a scientific measure to evaluate the performance of COVID-19 when both the number of confirmed and the number of recovered are considered output together. In addition, existing studies do not address the impact of environmental factors on efficiency, ignoring the impact of social distancing, health care policy, and socioeconomic factors in the various states. Previous studies regarding efficiency have focused on input contraction or output expansion when the operational efficiency level improves. However, it is inefficient when there is an undesirable output because efficiency measures should account for the simultaneous production of undesirable and desirable output, and DEA itself cannot determine the factors related to the efficiency. The combination of DEA and Classification and Regression Tree (CART) can resolve this problem by predicting the efficiency and uncovering the determinants of the various U.S. states’ efficiency under the COVID-19 pandemic scenario.

To analyze the factors that classify the efficiency and inefficiency of DMUs, the CART is the most transparent and comprehensible data-driven method. Breiman et al. [12] first introduced the CART algorithm as a hierarchical arrangement of decision nodes. A general classifier is constructed in the form of several splits on factors that separate a dataset into subgroups. The CART is good at detecting and accommodating interaction effects of different variables; however, it does not perform well in handling linear relations between variables. Therefore, researchers working on CART usually combine the results from Logistic Regression (LR) to overcome CART’s drawbacks. LR is a highly popular and powerful classification approach. It is an extension of linear regression as the outcome variable is categorical. The CART and LR have been applied in data analysis in many research studies [13,14,15,16].

Therefore, this paper will use DEA, CART, and LR to predict state COVID-19 response performance. Besides, Boosted Tree (BT) and Random Forest (RF) were applied to compare the model performance and to evaluate the importance of variables. The combined approach of CART, LR, BT, and RF will be henceforth referred to as machine learning (ML).

The rest of the paper is organized as follows: Section 2 deals with the methodology, Section 3 with results, Section 4 concludes.

## 2. Methodology

### 2.1. Data Description

Based on the availability of the dataset, the responsive performance of the U.S States COVID-19 was measured as an efficiency score. Fifty states were considered in this study. As a first step, referring to the literature, four inputs including the number of tested, public funding, number of healthcare employee, number of hospital beds, and the number of confirmed as an undesirable output, and number of recovered as a desirable output were collected for DEA analysis (see Table 1). The number of tested is the most important, affecting the country’s COVID-19 case [17]. The number of COVID-19 confirmed case is directly related to the number of tested. Public funding, health care employees and capacity of hospital beds are an essential input variable to measure performance because they are directly related to the number of recovered. The number of tested indicates the number of people tested for COVID-19, the number of confirmed indicates the number of COVID-19 positive cases, and the number of recovered indicates the patients recovered from COVID-19 were collected from Worldometer [18], where provide real-time Coronavirus updates. The number of confirmed, the number of recovered, and the number of tested starts in 1 March 2020 and last until 31 January 2021. There was a limitation to find the population density and health care employee during the COVID-19 period, we used the 2019 data for health care employee and hospital beds [4].

In the next stage, various COVID-19 related environmental factors were collected. To date, the United States has taken several actions to mitigate the COVID-19 and reduce barriers to testing and treatment for those affected. Based on the data providing state-level information in Kaiser Family Foundation (KFF) [19] and American Hospital Directory (AHD) [20], and Financial Industry Regulation Authority (FINRA) [21], this paper creates criteria for a different type of measure that affects states’ responsive performance. Table 2 shows three criteria measures and input abbreviation used in the ML analysis. We used the social distancing measure, including Population Density (PD), Large Gathering Ban (LGB), Non-Essential Business Closures (NEBC), Stayhome Duration (SHD), and Urban. PD, Urban, and SHD are quantitative variables. LGB and NEBC are categorical variables. With social distancing requirements in place for several weeks, U.S states have begun to roll back some social distancing policy by allowing some or all non-essential businesses to reopen, canceling stay at home orders and/or easing large gathering bans. Some levels of categorical variables for non-essential business closure ban were modified, including all with reduced capacity, some with reduced capacity, and no action. Large gathering bans were classified into three levels: lifted, below 25, and above 25. For the health policy measure, Expands Access to Telehealth Services (EATS), Mask Mandates (MASKM), Number of Tested per Population (NTP), Number of Vaccine Given per Population (NVGP), Total Hospital Beds per Population (THBP), and Public Funding per Population (PF) were collected. NTP, NVGP, THBP, and PF are quantitative variables, and EATS, MASKM are categorical variable that is indicated as Yes and No. Lastly, the socio-economic measure includes GDP, Health Care Employee per Population (HCEP), Physical Inactivity (PI), and Region (REG). GDP, HCEP, and PI are quantitative variables and REG is categorical variable.

### 2.2. The Hybrid Methodology of DEA and ML Frameworks

**Figure 1:**
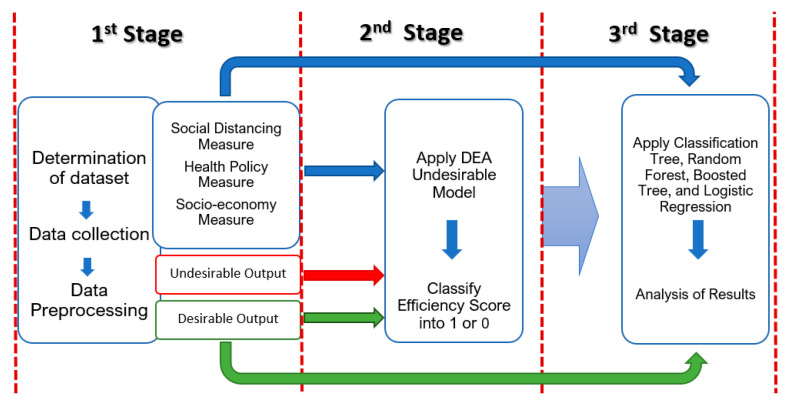
DEA and ML Methodology Frameworks.

This research applies a combined DEA, CART, and LR approach to uncover the determinants of efficiency of U.S. states under the COVID-19 situation so that our study reveals the impacts of different environmental factors on efficiency. All quantitative variables are normalized before running the model to deal with parameters of different units and scales in the first stage. In the second stage, authors apply DEA computation of the U.S. state’s performance and the efficiency scores obtained after running DEA with four inputs and one desirable output and one undesirable output. Each state is classified according to an efficient state (efficiency score of 1) and an inefficient state (efficiency score of less than one). Efficiency states are assigned as 1 and inefficient states are assigned as 0. They are used as a target for ML analysis. Fifteen environmental (exploratory) variables are used as inputs for Machine Learning. All computation is conducted in RStudio. Several packages are used such as “deaR” package for DEA, and “rpart”, “adabag”, “randomForest” are used for generating tree models, and “pROC”, “caret”, “e1071”, “recipes”, and “gains” are used for performance evaluations. “rpart.plot” and “RColorBrewer” are used for visualization. Figure 1 depicts our proposed hybrid model framework. In the following paragraphs, we explain the DEA and ML techniques used in our study.

### 2.3. Data Envelopment Analysis with Undesirable Output

As the COVID-19 pandemic creates both desirable and undesirable outputs, we must consider the impact of an undesirable output on the evaluation of the performance in terms of efficiency score. Seiford and Zhu [22] treated both desirable and undesirable outputs, providing a suitable tool for this research to evaluate COVID-19 efficiency with undesirable output. This research formulates a constant return to scale (CRS) DEA with undesirable output. The constraints of variable returns to scale (VRS) DEA with undesirable outputs [15]. Suppose the data matrix in DEA is expressed as:

(1) $$\left[\begin{array}{c} Y\\ -X \end{array}\right]=\left[\begin{array}{c} Y^{g}\\ Y^{b}\\ -X \end{array}\right]\;\;\;\;\;\;\;\;\;\;\;\;\;\;\;\;\;\;\;$$

where $Y^{g}$ is the desired output and $Y^{b}$ is the undesirable output. State wishes to increase $Y^{g}$ (e.g., Number of recovered from COVID-19) and to decrease $Y^{b}$ (e.g., confirmed cases of COVID-19) to improve performance. For our study, the DEA model developed by Charnes, Cooper, and Rhodes (CCR) [23] cannot be applied because CCR-DEA does not consider the undesirable output at all. Practically, we hope that our undesirable output decreases when the desirable output increases. Seiford and Zhu [22] modify the CCR-DEA model into a non-linear programming problem by still preserving the DEA model’s linearity and convexity. The monotone decreasing transformation method is used to treat the linearity by multiplying each undesirable output by −1. Then, they find a proper translation vector *w* to treat all negative values of undesirable output to be positive values. Therefore, the data matrix of (1) becomes as follows:

(2) $$\left[\begin{array}{c} Y\\ -X \end{array}\right]=\left[\begin{array}{c} Y^{g}\\ {\bar{Y}}^{b}\\ -X \end{array}\right]\;\;\;\;\;\;\;\;\;\;\;\;\;\;\;\;\;\;\;\;\;\;$$

where the *j*th column of undesirable output is ${\bar{y}}_{j}^{b}\;=\;-y_{j}^{b}+w>0$. Therefore, model (2) now becomes the following linear programming model:

(3) $$Max\;\;\;\;\theta \;s.t.\;\;\overset{n}{\underset{j=1}{\sum }}z_{j}y_{j}^{g}\;\geq \;hy_{o}^{g}\;\;\;\;\;\;\;\;\;\;\;\;\;\;\;\;\;\;\;\;\;\;\;\overset{n}{\underset{j=1}{\sum }}z_{j}{\bar{y}}_{j}^{b}\;\geq \;h{\bar{y}}_{o}^{b}\;\;\;\;\;\;\;\;\;\;\;\;\;\;\;\;\;\;\overset{n}{\underset{\;j=1}{\sum }}z_{j}x_{j}\leq x_{o}\;\;\;\;\;\;z_{j}\;\geq \;0,\;j\;=\;1,\;\ldots ,\;n$$

We solve the above linear programming model to obtain the state efficiency score of the CCR-DEA framework. Equation (3) provides the results of our main estimates that this study will use to combine with data mining techniques to predict the efficiency.

### 2.4. Classification and Regression Tree (CART)

In the second step, this study develops a CART where the independent variables represent the characteristics of observation and three measures described in Table 1. CART is known as non-parametric statistical method for predicting a dependent variable using some independent variable. This approach’s major goal is to uncover the predictive structure of COVID-19 efficiency in the U.S., thereby to create an accurate dataset. Therefore, the algorithm of CART permits, by binary recursive partitioning, to find all value of predictors, which minimize the weighted variance [24]. In CART, a root node includes all the observation and can be split into several child nodes. At the end, there are some terminals known as leaves. A set of observations is included in each leaf and an average of the dependent variable characterizes each leaf. Thus, the final tree is characterized by the dependent variable that is used for predicting independent variables. In the CART procedure, the same independent variable can be used on different levels of the tree. Once creating the tree, a pruning process is usually recommended to increase the predictive accuracy.

### 2.5. Logistic Regression

In our LR model, the dependent variable is in a categorical form and has two levels. Independent variables are in both numerical and categorical form. The following Equation (4) shows the binary multiple LR model [25].

(4) $$g\left(x\right)=\ln\left[\frac{\pi \left(x\right)}{1-\pi \left(x\right)}\right]=\ln\left[\frac{P\left(y=1|x\right)}{P\left(y=0|x\right)}\right]=\beta_{0}+\overset{p}{\underset{i=1}{\sum }}\beta_{i}x_{i}$$

Therefore, the log-likelihood function is used to estimate coefficients $\left({\left(\beta \right)}_{i}\right)$ in the model, which are calculated by iterative methods. The model’s odds ratio reflects the effect of the environmental factor on the COVID-19 efficiency.

### 2.6. Random Forest

RF is one of the multi-tree approaches that has been applied in various fields of study for classification [26,27]. The RF has been proven as a stable and effective classifier. It combines several decision trees and chooses the classification with the highest number of votes in the trees. Each different tree depends on the sampled random vector value from the same distribution for all trees in the forest. In RF, the margin of error depends on the strength of the trees’ different trees and the correlation between each tree.

### 2.7. Boosted Tree

Like RF, BT is a popular multi-tree approach that combines many decision trees to generate a robust classifier [28]. It builds a sequence of fitted trees so that each successive tree concentrates on correcting misclassified records from the preceding tree to reduce the misclassifications. The BT uses weighted votes to construct a final classifier, with higher weights given to later trees.

### 2.8. Performance Evaluation

This research uses multiple methods to evaluate the performance of ML model, which include confusion matrix, receiver operating characteristics (ROC) curves, lift chart, and area under the curve (AUC). A binary classifier is simply a classification model where the response has just two outcomes (efficient/inefficient state). Predictions made by a classification model give a probability from 1.0 to 0.0. However, the expected values recorded for each sample are binary (1 = efficient, 0 = inefficient). To convert the probability into binary class labels, the authors choose 0.5 to be the cutoff point for ML model [13]. In Table 3, four scenarios then described the difference between predicted and true binary outcomes as a confusion matrix which includes, True Positives (TP), False Positives (FP), False Negatives (FN), and True Negatives (TN). Sum of TP, FP, FN, and TN indicates the total number of samples (N).

The authors use the standard performance metrics to evaluate the accuracy of each ML model [29]. $\;Correct\;classification\;rate\;\left(c\right)=\frac{\left(TP+TN\right)}{N}$ defines the proportion of correctly identified samples of both binary classes. Then, the ROC curve is plotted to visualize the accuracy of predictions for a whole range of cutoff point. Two ratios such as sensitivity (or true positive rate), $se=\frac{TP}{\left(TP+FN\right)}$ and specificity (or true negative rate), $sp=\frac{TN}{\left(TN+FP\right)}$ are derived. A better model is when the ROC curve is closely plotted to the upper left corner. Moreover, the AUC value obtained from a ROC curve can be used to evaluate the model performance. AUC of 1.0 on the unit ROC space indicates a perfect prediction. Lastly, the lift chart is used to evaluate the performance of ML models. The lift chart helps to find the best predictive model among different models [30]. A better model results when the area between the lift curve and the baseline is greater.

## 3. Results

### 3.1. Descriptive Statistics

Table 4 display the descriptive statistics of variables, which are used in DEA analysis. For the undesirable output, the mean number of confirmed is found to be 503,330, while the mean number of recovered, a desirable output, is found to be 301,832. From the sample statistics, New York and New Jersey have the highest number of COVID-19 confirmed cases and Montana and Alaska have the lowest number of COVID-19 confirmed cases. In the case of the number of recovered, New York and Massachusetts have the highest number of COVID-19 recovered cases, and Alaska and Montana have the lowest number of COVID-19 recovered cases. The mean of input variables is also displayed in Table 4. From the sample data, New York and California have the highest number of tested and Wyoming and Vermont have the lowest number of tested. California and Texas have the highest number of healthcare employees, and Wyoming and Alaska have the lowest healthcare employees. California and Texas also have the highest hospital beds, while Vermont and Alaska have the lowest hospital beds. Lastly, a high amount of public funding related to health is allocated to California and Florida, while a lower amount of public funding is allocated to Vermont and Rhode Island. In the second stage of analysis, this paper examined potential determinants (environmental factors) of efficiency through ML analysis. Descriptive statistics of environmental factors are displayed in Table 5. Ten numerical and five categorical variables are considered to perform ML analysis.

### 3.2. DEA Results

Table 6 shows the summary of state efficiency scores. The results show that 23 states are efficient (efficiency score of 1), indicating that 46% of states are efficient in responding to COVID-19. The inefficient states’ efficiency scores ranged from 0.896 to 0.999, with Arkansas ranking first and Rhode Island ranking last among the inefficient states. The overall average efficiency score of inefficient state is 0.97, indicating that the states could produce, on average, 3% higher output with the same level of inputs. Based on the DEA efficiency score, states were divided into two groups for subsequent classification tree analysis: efficient (if efficiency score is equal to 1 and inefficient (if efficiency score is less than 1). We define this variable as state efficiency. State efficiency will be used as the outcome variable in the ML analysis.

**Figure 2:**
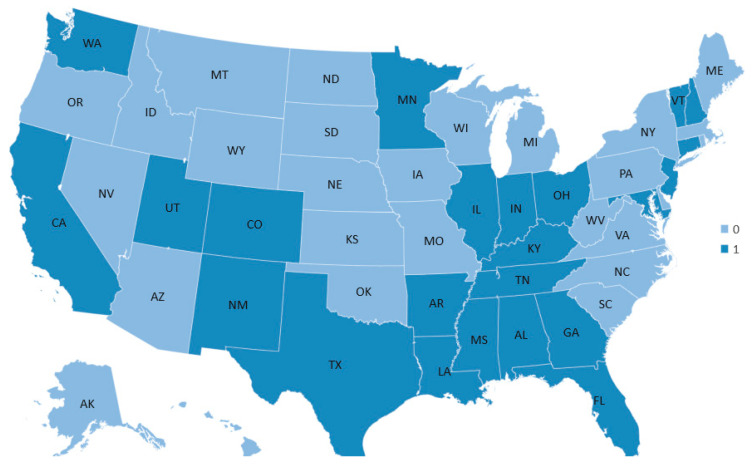
Efficient and Inefficient State (1 is Efficient state, 0 is Inefficient state).

Figure 2 depicts the distribution of efficient and inefficient states. As we can see, the dark blue color indicates efficient states and the light blue color indicates inefficient states. Efficient states are mainly located in the South, some in West and Midwest region. The result indicates that there may exist a potential relationship between region and efficiency. Thus, we add region as one of the environmental factors in the analysis.

### 3.3. Comparison of CART and LR

We use state efficiency as the outcome variable. As mentioned previously, input variables are PD, LGB, NEBC, SHD, Urban, EATS, MASKM, NTP, NVGP, THBP, PF, GDP, HCEP, PI, and REG. By using Bootstrap sampling method, the original dataset was increased to 220 records [31]. Then, we randomly partitioned the dataset into training (70% of records) and validation (30% of records). The training dataset is used to construct the CART and LR model.

**Figure 3:**
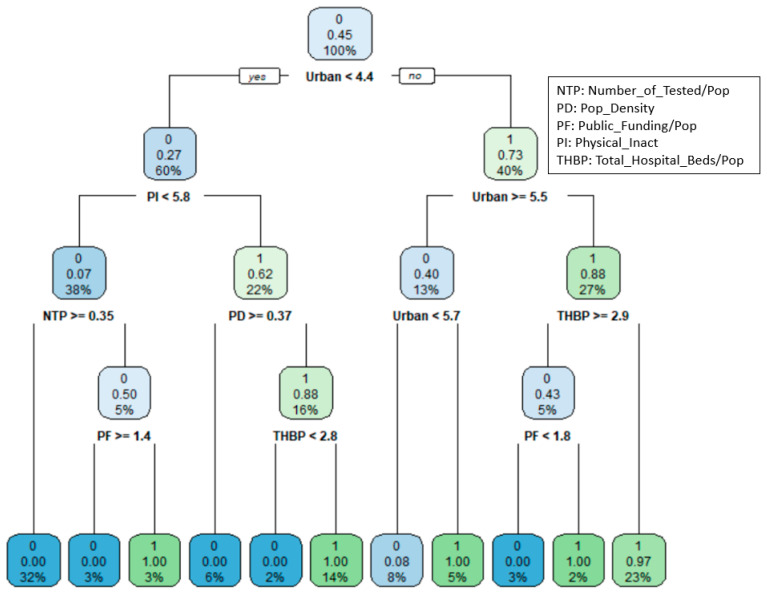
CART Results for States COVID-19 Responsive Performance.

Figure 3 shows the classification tree with 10 splits for the states’ COVID-19 efficiency. The top node includes all the records in the training data, of which 45% of states are efficient, and 55% of states are inefficient. The “0” in the top node’s rectangle denotes the majority group is inefficient (0 = inefficient). The first split is on Urban. A state with Urban percentage less than 4.4 will go to the left child node and a state with Urban percentage greater than or equal to 4.4 will go to the right child node.

The next split is on PI for states with Urban percentage less than 4.4 and Urban percentage for state with Urban percentage greater than equal to 4.4. The terminal node shows the final classification for states. Among the 11 terminal nodes, five lead to the classification of “efficient” and six lead to “inefficient” classification. From the classification tree the most important variable for efficiency is the Urban. State with Urban percentage less 4.4, PI less than 5.8, and NTP greater than or equal to 0.35 are considered as inefficient states. If Urban percentage is less than 5.5 but greater than or equal to 4.4 and THBP is less than 2.9, then state is considered as efficient. In overall, Urban, PI, NTP, PD, THBP, and PF directly affect the classification of the states.

**Figure 4:**
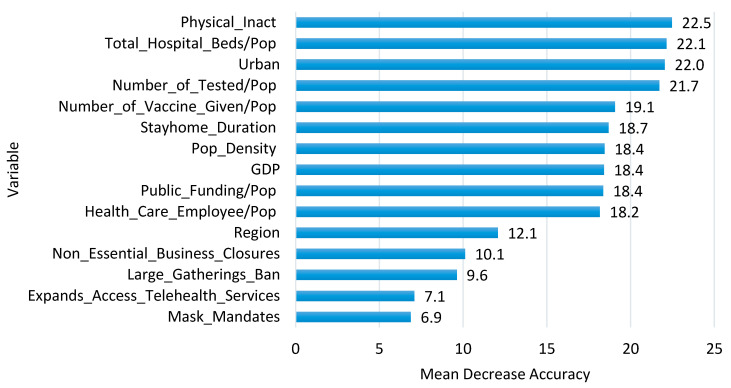
Importance Score of Predictor Variables from RF (Mean Decrease Accuracy).

Figure 4 shows the importance of predictor variables generated from the RF model. As can be seen in the figure, PI has the highest mean decrease accuracy score (22.5), followed by THBP (22.1), Urban (22.0), NTP (21.7), which are the most important variables for predicting state efficiency. It shows similar findings in Classification Tree (Figure 3) in that Urban, PI, THBP, NTP, PD are top priorities in classifying state efficiency levels. On the other hand, MASKM (6.9) and EATS (7.1) shows the lowest mean decrease accuracy score, which are the least important variables for predicting state efficiency, meaning that we might not need to retain all of the environmental factors to create a predictive model.

LR is another method for predicting the efficiency of the categorical outcome variable. Efficiency is predicted based on a number of environmental factors. Table 7 shows the odds ratios (OR) and *p*-value of the different significance levels. For the environmental factor of NEBC, two dummy variables are added when constructing a LR model, and All with Reduced Capacity is considered as a reference category. The odds of being efficient for NEBCNo is 0.0004, which indicates that No action is less likely to be efficient than the All with Reduced Capacity holding all other variables as constant.

For the EATS factor, No is considered as a reference category. The odds of being efficient for Yes is 57.5526, which indicates that Yes increases the likelihood of efficiency than No. For REG, three dummy variables are added, and the Midwest is considered as a reference category. The odds of being efficient for REGNortheast is 0.0856, which indicates that Northeast is less likely to be efficient than the Midwest. Both South and West Regions decrease the likelihood of efficiency. Take GDP as an example to explain the quantitative variable. For one unit increase in GDP, the odds of being classified as an efficient state increase by a factor of 4.5465.

Considering the coefficients’ statistical significance (*p*-value < 0.05), PD, LGBLifted, NEBCNo, NEBCSome, SHD, Urban, EATSYes, MASKMYes, NTP, NVGP, THBP, GDP, and PI are statistically significant to predict the efficiency. On the other hand, LGBBelow25, PF, HCEP, REGNortheast, REGSouth, and REGWest were not related to efficiency. These factors are consistent with factor importance ranking obtained from RF (see Figure 4).

Among the 15 considered environmental factors, twelve factors (original categorical variables are considered) affect efficiency significantly in the LR model, and six factors are effective in constructing an optimal classification tree. Five environmental factors found significant in the LR model were also found significant in the classification tree.

### 3.4. Performance Evaluation

We used a confusion matrix to evaluate the performance of different models. Table 8 summarizes the correct and incorrect classifications for four different models using training and validation data.

For the classification tree, out of 154 cases of training data, 69 cases are predicted to be efficient with an accuracy of 99% and 83 cases are predicted to be inefficient with an accuracy of 98.81%. The overall accuracy is 98.70%. For the validation data, 66 cases are considered in which 27 cases are correctly classified as efficient and 33 cases are correctly classified as inefficient. The efficiency accuracy rate is found to be 96.43% and the inefficiency accuracy rate is found to be 86.84%. The classification tree’s overall accuracy level is 90.91%, which indicates a relatively high level of confidence.

For BT and RF, the training data has an estimated accuracy rate of 100%. The accuracy rate reduces to 95.45% for the validation data. The efficiency and inefficiency accuracy rates are 100% and 92% respectively, which indicates that these two models are good at classifying efficient and inefficient states.

From the result by LR model, out of 154 cases of training data, 55 cases are predicted to be efficient with an accuracy of 78.57% and 74 cases are predicted to be inefficient with an accuracy of 88.10%. The overall accuracy is 83.77%. For the validation data, 66 cases are considered in which 22 cases are correctly classified as efficient and 32 cases are correctly classified as inefficient. The efficiency accuracy rate is found to be 78.57% and inefficiency accuracy rate is found to be 84.21%. The overall accuracy level for the LR is 81.82%.

**Figure 5:**
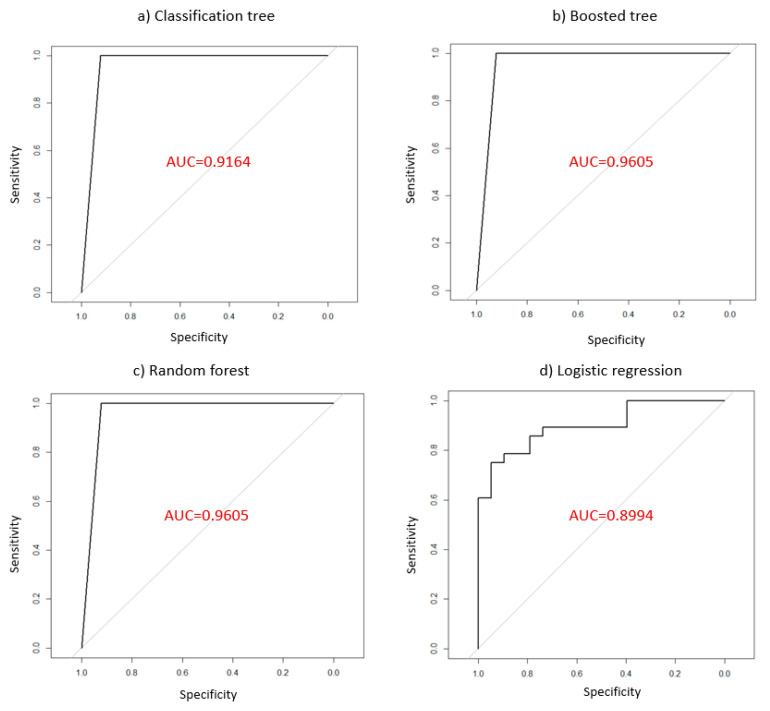
ROC curve for four model’s accuracy.

**Figure 6:**
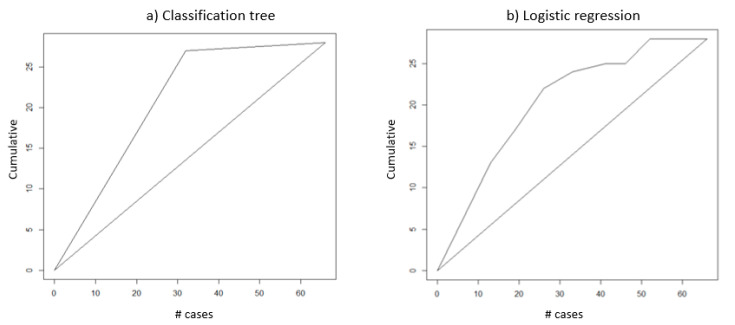
Lift Chart for Validation Data.

Figure 5 shows the ROC curves for the four different models. The ROC curve plots the different pairs of specificity and sensitivity as the cutoff value decreases from 1 to 0. Area under the curve (AUC), which ranges from 1 (perfect prediction) to 0.5 (random coin flipping), is used to measure the model’s performance. AUC is found to be 0.9164 for CART, 0.9605 for BT, 0.9605 for RF, and 0.8994 for LR. From this measure, BR and RF are better methods to predict the efficiency than CART and LR. However, CART is better than LR. Meanwhile, BT and RF are combined from the results of multiple trees and they allow for better consistency of results and robustness of predictions. They usually perform better than a single tree. However, their result cannot be displayed in a tree-like diagram. An RF and BT aka “black-box model” is less interpretable than a single classification tree. Therefore, we only compared lift chart for classification tree and logistic regression in Figure 6.

The lift chart helps visualize for measuring model performance, which is the ratio between results obtained from the predictive and random classification models. The greater the area between the baseline and the lift curve, the better the model.

As it can be seen from Figure 6, if the top 30 records are selected, CART can correctly classify 25 records (83%) and the LR model can correctly classify 23 records (77%). If the top 40 records are selected, CART can correctly classify 27 records (68%) and the LR model can correctly classify 25 records (63%). From the analysis result, CART performs better than LR and these two models are much better than random classification.

## 4. Conclusions

The application of DEA, CART, and LR on measuring the COVID-19 response performance of states gives a new angle to fight against the Coronavirus outbreak. From DEA analysis, the finding indicated that 23 out of 50 states were efficient in responding to COVID-19. As a second stage, CART and LR are used to find the associated relationship between efficiency and 15 environmental variables. Variables are categorized into three measurement groups: social distancing measure, health policy measure, and socioeconomic measure to run four ML models.

CART identified the six key predictors: Urban, PI, NTP, PD, THBP, and PF. RF and BT were extensions of a single classification tree to improve the classification tool’s robustness. RF produced variable importance scores, which show the measure of relative contribution of the different environmental factors. From the importance of predictor variable analysis, using RF, PI, THBP, Urban, NTP, and NVGP were among the top 5 priorities in classifying the efficiency. Lastly, researchers used the LR approach to predict the efficiency for comparison with our other proposed models. Five environmental factors such as Urban, PI, NTP, PD, and THBP being found significantly in LR model were also found significantly in CART.

Our study is well justified by assessing the performance of different model’s confusion matrix, ROC curve, and lift chart. BT and RF were better predictive models because of higher accuracy rate and higher AUC value. Moreover, CART performed better than LR. Although DEA can explain the efficiency scores, but it cannot explain the environmental factors related to the inefficiency of the DMUs. Therefore, the CART and LR approaches help explain the efficiency results obtained by DEA by observing the environmental factors associated with efficiency and inefficiency.

This study’s results may be of interest to health care decision-makers who are involved in COVID-19 response management planning and wish to maximize the statewide performance.

First, health care decision-makers in government and industry need to incorporate the results of this study, which focus on social distancing measures, health policy measures, and socioeconomic measures in addition to experimental results. The functioning of the COVID-19 pandemic may be encumbered by increasing health care provider capacity that may be difficult to expand based on current capacity level. COVID-19 spreads rapidly to urban areas, where higher population density exists. Moreover, states with high populations tend to be efficient; thus, paying attention to the rural areas with states with lower populations is important to improve the efficiency. States could allocate additional health care resources such as a health care employee and hospital bed by establishing a resource consortium program between states for effective utilization of health care resource. States should also establish effective vaccine allocation program to maximize the vaccine supply. Knowing the factor affecting the efficiency will be very important for health care policy makers to establish the COVID-19 responding policy for each state. Our results might be the set of rules that can be used for health care policymakers to improve statewide pandemic response performance.

Secondly, health care decision-makers can supplement our study results by conducting window analysis of states’ operational efficiency. One limitation of this study is that detailed panel data for some of the variables, such as number of health care employees, population density, GDP, and public funding, were not publicly available. Usually, they were aggregated into one year. The limitation of the study can be overcome if disaggregated monthly or weekly data is available. These are important parameters that health care decision-makers should include when modeling the determinants of efficiency in COVID-19 or similar unprecedented pandemic situation in the future. Our study has taken one step towards evaluating the proper mechanisms that can help U.S. governments improve their efficiency regarding COVID-19. Future research should find out which measure would work best in this context. Moreover, this study used a bootstrapping sampling method to tackle our issue. We think that use of county-level to predict the efficiency would be a good idea, but this is beyond the scope of this paper.

## Figures and Tables

**Table 1:** Input and output variables used in DEA analysis.

Input/Output	Variable
Undesirable Output	Number_of_confirmed (in person)
Desirable Output	Number_of_Recovered (in person)
Input	Number_of_Tested (in person)
	Health_care_employee (in person)
	Total_Public_Funding (in dollar)
	Total_Hospital_Beds (in unit)

**Table 2:** Three Input Criteria and Input Abbreviation used in the ML Analysis.

Criteria	Input Variable	Input Abbreviation
Social Distancing Measure	Pop_Density ^a^	PD
	Large_Gatherings_Ban	LGB
	Non_Essential_Business_Closures	NEBC
	Stayhome_Duration ^b^	SHD
	Urban ^c^	Urban
Health Policy Measure	Expands_Access_Telehealth_Services	EATS
	Mask_Mandates	MASKM
	Number_of_Tested/Pop ^d^	NTP
	Number_of_Vaccine_Given/Pop ^e^	NVGP
	Total_Hospital_Beds/Pop ^f^	THBP
	Public_Funding/Pop ^g^	PF
Socioeconomic Measure	GDP	GDP
	Health_Care_Employee/Pop ^h^	HCEP
	Physical_Inact ^i^	PI
	Region	REG

^a^ Measured by people per square mile in each state. ^b^ Stay Home Duration is calculated based on order date and order expiration date for each state (in day). ^c^ Urban is the percent of the U.S. population living within urban areas. ^d^ Total number of COVID-19 tested in each state is divided by total population. ^e^ The percentage of COVID-19 vaccine given in each state. ^f^ Total number of hospital beds available in each state is divided by total population. ^g^ The state and federal dollars allocated to public health and states per person (in dollar). ^h^ Total number of health care employees is divided by total population in each state. ^i^ The percentage of adults who have done no physical activity other than their regular job in the past 30 days.

**Table 3:** The Confusion Matrix of ML model.

Count	True Efficient State	True Inefficient State
Predicted efficient state	TP	FP
Predicted inefficient state	FN	TN

**Table 4:** Descriptive Statistics of the Dataset used in DEA.

Variable		Mean	Std.dev	Min	Max	Sum
Undesirable Output	Number_of_confirmed (in person)	503,330	583,039	11,165	3,199,895	25,166,517
Desirable Output	Number_of_Recovered (in person)	301,832	366,394	5901	1,859,898	15,091,582
Input	Number_of_Tested (in person)	5,956,730	7,628,919	390,763	40,688,908	297,836,475
	Health_care_employee (in person)	334,719	354,014	25,410	1,670,370	16,735,960
	Total_Public_Funding (in dollar)	571,088,722	747,072,557	63,506,800	4,552,873,746	28,554,436,123
	Total_Hospital_Beds (in unit)	14,951	15,608	1046	74,286	747,572

**Table 5:** Descriptive Statistics of Environmental Factors used in ML Analysis.

Numerical Variables	Mean	Std.dev	Min	Max	Sum
Public_Funding/Pop	99.16	44.57	46.00	281.00	4958.00
Health_Care_Employee/Pop	0.052	0.008	0.034	0.070	2.615
Physical_Inact	24.08	3.84	16.40	32.40	1204.00
GDP	61,880.34	11,605.49	40,464.00	90,043.00	3,094,017.00
Urban	73.59	14.57	38.70	95.00	3679.30
Stayhome_Duration	35.72	18.33	-	71.00	1786.00
Total_Hospital_Beds/Pop	0.002	0.001	0.001	0.004	0.121
Number_of_Tested/Pop	0.29	0.41	0.02	2.16	14.50
Pop_Density	203.90	267.36	1.00	1215.00	10,195.00
Number_of_Vaccine_Given/Pop	0.069	0.016	0.050	0.132	3.470
**Categorical Variables**	**Levels of Category**	**Description**			
Region	4	South, West, Northeast, and Midwest			
Non-Essential Business Closures	3	All with Reduced Capacity, Some with Reduced Capacity, and No action			
Large Gatherings Ban	3	Lifted, Below 25, and Above 25			
Expands Access Telehealth Services	2	Yes or No			
Mask Mandates	2	Yes or No			

**Table 6:** Summary of state efficiency scores.

State	Efficiency Score	State	Efficiency Score	State	Efficiency Score
Alabama	1	Ohio	1	Michigan	0.948
Arizona	1	Tennessee	1	Nebraska	0.946
California	1	Texas	1	South Dakota	0.944
Colorado	1	Utah	1	North Dakota	0.942
Connecticut	1	Virginia	1	North Carolina	0.941
Florida	1	Washington	1	Montana	0.941
Georgia	1	Arkansas	0.999	New Hampshire	0.933
Indiana	1	Illinois	0.991	Wyoming	0.931
Iowa	1	Wisconsin	0.990	Maine	0.929
Kentucky	1	Oklahoma	0.990	Kansas	0.926
Louisiana	1	Oregon	0.988	Vermont	0.924
Maryland	1	Mississippi	0.987	Idaho	0.922
Minnesota	1	Hawaii	0.978	Alaska	0.918
Missouri	1	Massachusetts	0.968	Delaware	0.911
New Jersey	1	West Virginia	0.968	Pennsylvania	0.899
New Mexico	1	South Carolina	0.950	Rhode Island	0.896
New York	1	Nevada	0.950		

**Table 7:** The results of fitting a logistics regression model on efficiency.

	Environmental Factors	OR	*p*-Value	
Social Distancing Measure	PD	0.1595	0.0101	*
	LGBBelow25	3.2483	0.1551	
	LGBLifted	7.5648	0.0354	*
	NEBCNo	0.0004	0.0001	***
	NEBCSome	0.0942	0.0094	**
	SHD	0.0118	0.0003	***
	Urban	14.4586	0.0116	*
Health Policy Measure	EATSYes	57.5526	0.0015	**
	MASKMYes	8.3107	0.0185	*
	NTP	0.0607	0.0012	**
	NVGP	3.1527	0.0101	*
	THBP	0.0407	0.0011	**
	PF	1.6053	0.2534	
Socio-Economic Measure	GDP	4.5465	0.0355	*
	HCEP	1.8354	0.4298	
	PI	38.5216	0.0002	***
	REGNortheast	0.0856	0.0906	
	REGSouth	0.6671	0.7627	
	REGWest	0.2415	0.4932	

* *p* < 0.05, ** *p* < 0.01, *** *p* < 0.001.

**Table 8:** The accuracy results for the four different methods.

		Training Data		Validation Data	
		Efficiency	Inefficiency	Efficiency	Inefficiency
**Classification Tree**	Accuracy rate	99%	98.81%	96.43%	86.84%
	Average	98.70%		90.91%	
**Boosted Tree**	Accuracy rate	100%	100%	100.00%	92%
	Average	100%		95.45%	
**Random Forest**	Accuracy rate	100%	100%	100.00%	92%
	Average	100%		95.45%	
**Logistic Regression**	Accuracy rate	78.57%	88.10%	78.57%	84.21%
	Average	83.77%		81.82%	

## Data Availability

The excel datasets generated during and/or analyzed during the current study are available from the corresponding author on reasonable request.
